# MicroRNA Expression in β-Thalassemia and Sickle Cell Disease: A Role in The Induction of Fetal Hemoglobin

**DOI:** 10.22074/cellj.2016.3808

**Published:** 2016-01-17

**Authors:** Najmaldin Saki, Saeid Abroun, Masoud Soleimani, Maria Kavianpour, Mohammad Shahjahani, Javad Mohammadi-Asl, Saeideh Hajizamani

**Affiliations:** 1 Health Research Institute, Research Center of Thalassemia and Hemoglobinopathy, Ahvaz Jundishapur University of Medical Sciences, Ahvaz, Iran; 2 Department of Hematology, Faculty of Medical Sciences, Tarbiat Modares University, Tehran, Iran; 3 Department of Medical Genetics, School of Medicine, Ahvaz Jundishapur University of Medical Sciences, Ahvaz, Iran

**Keywords:** MicroRNAs, β-Thalassemia, Sickle Cell Disease, Fetal Hemoglobin

## Abstract

Today the regulatory role of microRNAs (miRs) is well characterized in many diverse cel- lular processes. MiR-based regulation is categorized under epigenetic regulatory mecha- nisms. These small non-coding RNAs participate in producing and maturing erythrocytes, expressing hematopoietic factors and regulating expression of *globin* genes by post-tran- scriptional gene silencing. The changes in expression of miRs (miR-144/-320/-451/-503) in thalassemic/sickle cells compared with normal erythrocytes may cause clinical severity. According to the suppressive effects of certain miRs (miR-15a/-16-1/-23a/-26b/-27a/-451) on a number of transcription factors [myeloblastosis oncogene (*MYB*), B-cell lymphoma 11A (BCL11A), *GATA1*, Krüppel-like factor 3 (*KLF3*) and specificity protein 1 (*Sp1*)] during *β globin* gene expression, It has been possible to increasing *γ globin* gene expression and fetal hemoglobin (HbF) production. Therefore, this strategy can be used as a novel therapy in infusing HbF and improving clinical complications of patients with hemoglobi- nopathies.

## Introduction

MicroRNAs (miRs) are a group of non-coding RNAs of ~22 nucleotides in length, which post-transcriptionally regulate the expression of their target genes as well as chromatin-remodeling, proliferation, differentiation and apoptosis (1,2). These single stranded molecules form a miRNA-mediated silencing complex (miRISC) complex with other proteins which bind to the 3´ untranslated region (UTR) of their target mRNAs so as to prevent their translation in cytoplasm (3). They have a great impact on cell functions in this way (4), and are thought to regulate homeostatic and pathologic conditions of various diseases, particularly hematologic, infectious and endometrial disorders as well as different types of cancer. 

Hemoglobinopathies are the most common type of blood disorders caused by genetic mutations in human *β globin* gene (*HBB*) that result in abnormal hemoglobin structure. Sickle cell disease (SCD) and β-thalassemia are considered as two prevalent forms of these disorders (5). Increasing fetal hemoglobin (HbF) is a significant therapeutic tool to overcome anemia and ineffective hematopoiesis. This type of Hb has high levels in the fetus and produced at low level in some adults (6). By balancing the ratio of pathological chains of α/β, the accumulation of α globin chains in erythroid precursors is reduced and thus inhibits ineffective erythropoiesis, therefore improving the oxygen supply to tissues and relieving clinical symptoms (7,8). 

Different approaches including hydroxyurea, epigenetic modifications (e.g. inhibition of *γ globin* gene promoter methylation or deacetylation with thalidomide and sodium butyrate) and miR-based regulation (miR-15a/-16-1/-486-3p expression changes) are used for induction of γ globin which may be used for therapeutic purposes in SCD and β-thalassemia patients (9-14). In this review, we discuss changes of miR expression in β-thalassemia and SCD as two common hemoglobinopathies and also illustrate their roles in expression of globin chains so as to introduce new therapies for patients by inducing HbF.

## Dysregulation of microRNA expression in β-thalassemia

Thalassemia is a hereditary blood disorder, caused by more than 200 autosomal mutations in *globin* genes resulting in a failure to produce normal globin chains, which shows different phenotypes due to severity of anemia and clinical complications (15,16). α and β-thalassemia resulting from different mutations in the α and *β globin* genes respectively, lead to chronic anemia and ineffective erythropoiesis in these patients (17). In β-thalassemia, excess α chains accumulate in erythrocytes due to insufficient expression of β globin chains inducing hemolysis and ineffective hematopoiesis. Repeated blood transfusions and HbF synthesis are ways to achieve therapeutic goals in these patients (18). Given that miRs are involved in the expression of *globin* genes and also transcriptionally regulate erythroid-specific genes [e.g. Kruppel-like transcription factor D (*KLFD*)], it can be envisaged that changes in expression of these small RNAs is effective in reducing clinical complications in thalassemic patients (19). 

**Fig.1:**
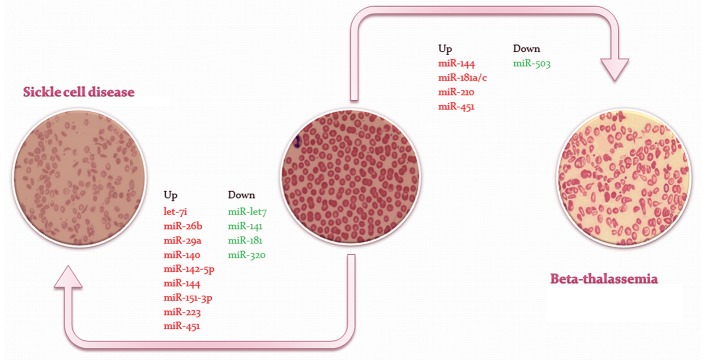
Dysregulation of microRNA expression in β-thalassemia and sickle cell disease. In this figure the microRNAs involved in β-thalassemia and sickle cell disease has been depicted. According to upor down-regulation of these miRs, the erythrocyte fate is determined. Some of these are erythrocytes specific microRNA as mir-451 with increased expression during the differentiation of this lineage. miR; MicroRNA.

Accumulation of a globin chains destroy the erythrocyte membrane in thalassemic cells (20). Most of miRs which inhibit α gene expression, improve hemolytic anemia. MiR-144 as a erythroid-specific miR, prevents cell lysis with direct targeting of erythroid-specific *KLFD* (21). *KLFD*, by interacting with *CACCC* sites in miR-144 and the *α globin* gene promoter, acts as a co-regulator of both genes (21,22). It has been shown that the level of miR-144 expression negatively controls *α/β globin* gene expression in children with β-thalassemia major. This regulation of gene expression provides the primary basis of β-thalassemia major treatment in which preventing accumulation of excessive α globin may reduce clinical complications in patients with thalassemia (23). MiR-150 is another candidate for suppressing the *α globin* gene expression. This miR has various roles in erythroid, lymphocyte and megakaryocyte cell types. Although it has reduced expression during erythroid differentiation, it shows much lower amounts in polycythemia vera. Control of erythroid progenitor cell fate as well as suppression of *α globin* gene expression are other functions of this miR along with targeting myeloblastosis oncogene (*MYB*) (Fig .1) (24,25). 

Chronic anemia, the most significant factor in hypoxia induction, causes over-expression of hypoxic-dependent miRs known as hypoxamirs in thalassemia patients (26,27). miR210, a hypoxamir, increases in normal erythroid progenitor cells and β-thalassemia/HbE and also enhances *GATA1*, Erythroid Kruppel-like factor (*EKLF*) and erythroid-specific isoform of 5-amino-levulinate synthase 2 (*ALAS2*) regulatory factors in hypoxia-induced erythropoiesis (26). This miR also affects the expression of transferrin receptor (CD71) and glycophorin A (GPA, CD235a) during erythroid differentiation thus increasing levels of α and γ globin followed by raised HbF levels (26,28). High level production of HbF, as a result of alteration in *globin* gene expression, is a therapeutic approach in hemoglobinopathy patients. 

Decreased expression of miR-503 which regulates cell cycle arrest and apoptosis, was found in cells with β-thalassemia mutations (29). Cell division cycle 25A (CDC25A), a target gene for this miR, is involved in cell cycle and DNA damage responses (30). Thalassemic cells show 1000-fold CDC25A over-expression due to low levels of this miR which is an important factor for ineffective hematopoiesis occurring in such patients (29). Induction of miR-503, miR322/424 or other factors which are dependent on hypoxia such as miR-21 and cyclin-dependent kinase inhibitor p21, suppress CDC25A and prevent growth in erythroid progenitor thalassemic cells and cancer cells respectively (29,31,32). 

Another erythroid-specific miR is miR-451 which induces erythroid differentiation from CD133+ cells and also during erythropoiesis, has extra expression (33,34), however, in β-thalassemia/HbE cells, miR-451 is significantly over-expressed and is associated with ineffective hematopoiesis, chronic hemolytic anemia and generally thalassemia severity. Over expression of miR-451 is associated with decreasing levels of α chain, glycophorin-A and GATA1 transcripts and is also observed in thalassemic cells which have lower hemoglobin levels and more reticulocytes (35). MiR-451 over expression is more effective on raising expression of *α* and *β globin* genes than γ globin (34). Increased expression of this miR is also present in other hemoglobinopathies and polycythemia vera where erythropoiesis takes place at a higher level (36,37). 

Overall, according to the significance of miRs in controlling expression of *globin* genes, reactivation of these genes, which may change the status of thalassemic cells and improve the pathophysiology and clinical symptoms of hemoglobinopathies, would make it possible to use these small non-coding RNA as new therapeutic targets. 

## Dysregulation of microRNA expression in sickle cell disease

SCD is a common disorder of the *HBB* that is created by a single amino acid substitution in the sixth position of this gene. The produced HbS polymerize in de-oxygenated conditions inside the cell (38). In a survey of miR expression profiles in reticulocytes, and normal and sickle mature erythrocytes a considerable difference that can be effective in the severity of anemia and oxidative stress conditions was demonstrated (39). Analysis of the effect of miR expression changes on HbSS erythroid resistance against Plasmodium falciparum led to the observation that level of miR-451, let-7i and miR-223 is higher in HbSS cells than HbA and are thus helpful in the reduction of parasite infection (40). Transfection of these miRs considerably reduced the parasitemia rate due to the induction of translation inhibitors of mRNA including parasite’s cAMP-dependent protein kinase (PKA-R), phosphoethanolamine Nmethyltransferase (PEAMT), and the 28S and 18S rRNAs and reduction of parasite growth (36,41). Studies have shown that inhibition of the translocation of miR-451 and miR-223, by using 2´-O-methyl antisense oligonucleotides, reduced sickle cell resistance to malaria. This suggests that the induction of these miRs in infected cells can be used for host cell defense against pathogens (41). 

CD71 as transferrin receptor plays an important role in the process of iron absorption and the terminal differentiation of erythroid cells. Posttranscriptional regulation of this CD marker is controlled by miRs like miR22/-200a/-320 (42). Previous studies on miR320 have shown that this miR hybridizes to the 3'UTR of the CD71 transcript and represses its translation. Since this CD71 is a marker of erythropoiesis and its over-expression is reported in many malignancies, inhibition of its expression by miR-320 could be used as a new treatment for cancers with high proliferation rate and reduced iron levels, and also reduce the pace of cell cycle progression (36). Due to decreased expression of miR-320 in HbSS cells, CD71 is over-expressed on the surface of sickle reticulocytes indicating a defect in erythropoiesis induced by hemolysis. The importance of miR-320 in sickle cells is apparent by dysregulated maturation and decreased cell survival (Fig .1) (39) . 

Nuclear related factor 2 (*NRF2*) is the main factor in oxidative stress response and cellular antioxidant defense system (43). The reduction of *NRF2* leads to decrease the cellular glutathione levels and the cells will be more sensitive to oxidative stress, miR-144 contributes in the process through *NRF2* targeting. Higher levels of miR-144 expression in HbSS reduce the level of NRF2 and indeed diminish oxidative stress tolerance in these cells. Over expression of miR-144 is associated with severity of anemia and decreased hemoglobin/hematocrit cell count, and also leads to the lack of antioxidant proteins such as glutamate-cysteine ligase, catalytic/modifier subunit (GCLC/M) and superoxide dismutase 1 (SOD1) (44). The miR144/NRF2 regulatory mechanism predisposes HbSS to oxidative stress, hemolysis and more severe anemia. Therefore, erythrocyte miR expression manipulation provides a new approach to reduce clinical and pathological signs in SCD patients (39). 

Therapies for SCD patients include inducing HbF synthesis using hydroxyurea, butyrate, 5-azacytidine and currently decitabine (45,46). Based on the discussion above, some miR have the potential to induce HbF synthesis in primary erythroid cells, therefore replacing methods that use drugs to avoid the side effects and improve clinical signs of the patients. 

## Role of microRNAs in *γ globin* gene expression and fetal hemoglobin level

The hemoglobin tetramerous molecule is made of two different chains, α and β globin chains which are encoded by genes located on chromosomes 16 and 11 respectively. The α gene cluster contains genes *ζ2, α1, α2* and several pseudo genes. The *β* gene cluster includes *γ, β, δ , ε *genes and a pseudo gene (6,47). The expression of the *β* gene cluster is regulated by an important cis-element, locus control region (LCR) in the five DNase I hypersensitive sites upstream of the *ε globin* gene and also rearrangement of these genes at the stage of hematopoiesis (8,48). 

During evolution, two types of *globin* gene switching occur. The first is when the γ gene is replaced by ε and hence the production of HbF (α2γ2) in the fetal liver. The other switching occurs for replacement of the *β* gene instead of γ, resulting in reduced expression of HbF (48,49). In many hemoglobinopathies, different drugs can induce HbF to achieve the benefits of HbF expression and decrease abnormal HbA expression. Hemoglobin expression is adjusted by posttranscriptional modifications, several transcription factors bind to cis-acting DNA elements and increase expression of *γ globin* gene (50). 

**Fig.2:**
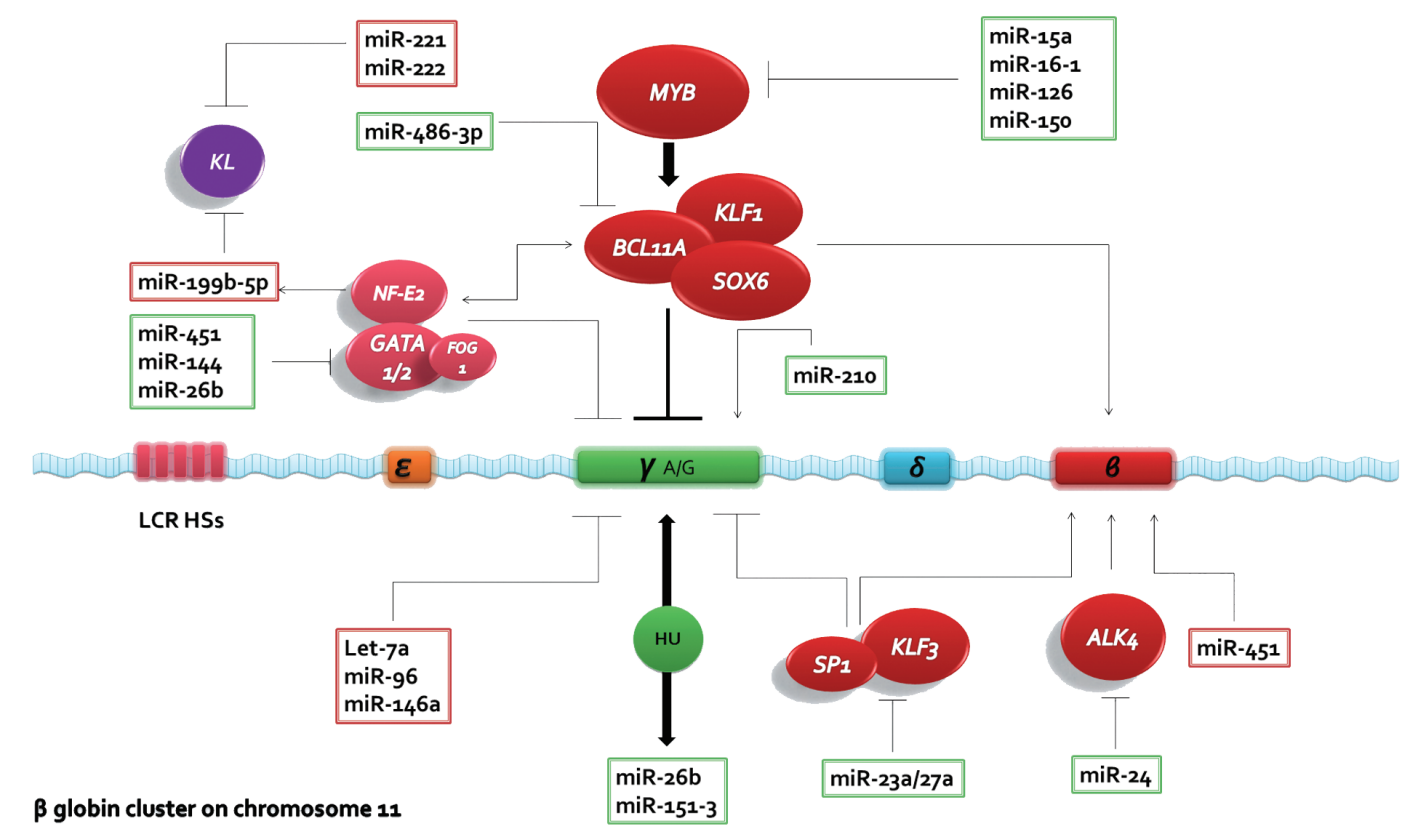
Molecular regulation of the fetal to adult hemoglobin switch with regulatory microRNAs. Human *β globin* gene locus located on chromosome 11, containing 4 functional genes that are expressed during different stages of development. Gene ε, the globin expression in the early weeks of gestation, the γ gene expression in the fetal stage is responsible for the production of HbF, β and δ genes code globin in the adult after birth. Four major transcription factors in switching γ to β are KLF1, BCL11A, *MYB* and SOX6 which can cause silencing of *γ* and *β genes*. Sp/KLF3 are also important transcription factors in β globin expression that have an inhibitory effect on the expression of γ globin. KL influences erythropoiesis and also *globin* genes switching but its function is inhibited via miR-221/-222 and miR-199b-5p induced by the NFE2/GATA1 complex. GATA 1/2, FOG 1 bind to globin locus and inhibit the γ globin expression. HU drug used to increase fetal hemoglobin, which directly increases miR-26b/-151-3. ALK4 participates in erythroid differentiation and maturation and increases *β globin* expression. MiRs mentioned have the ability to inhibit of each one of the factors. Those increasing and decreasing γ globin expression are shown in green and red boxes respectively. ALK4; Activin type I receptor, BCL11A; B-cell lymphoma 11A, FOG1; Friend of GATA1, GATA1/2; GATA binding protein 1/2, HU; Hydroxyurea, HSs; DNase I–hypersensitive sites, KLF1; Krüppel-like factor 1, KL; Kit receptor ligand, LCR; Locus control region, NFE2; Nuclear factor, erythroid 2, Sp; Specificity protein and miR; MicroRNAs.

The major transcription factors in switching γ globin to β globin are Krüppel-like factor 1 (KLF1), B-cell lymphoma 11A (BCL11A) and MYB (Fig .2). These factors either directly or indirectly are responsible for regulating γ gene silencing. Induction of γ globin repressor and cell cycle during erythropoiesis, and changes in the expression of these factors can be effective for indirect synthesis of HbF (51,52). This approach could be used to induce HbF in patients with β globin disorders (53). 

In the differentiation and development of mature erythrocytes from hematopoietic stem cells, miRs are expressed differently depending on the stage of differentiation and are also effective on important transcription factors in the production of HbF. For example, miR-451 expression becomes higher during the differentiation of erythroid cells while expression of miR-150/-155/-221/-222 decrease (Table 1) (35) . 

Observational and clinical studies suggest that increased HbF in patients with thalassemia and SCD can reduce severity of these diseases for the duration of years under investigation and trying different approach for increase the γ chain expression and production of HbF. Generally treatment of these patients is done with the drug hydroxyurea which increases expression of miR-26b/-151-3p/-148a/-494 of which only miR-/26b and miR-151-3p are hydroxyurea-mediated HbF inducing agents (54). 

Comparison of miR expression in the cord blood and adult blood reticulocytes has shown many differences in the expression levels of miR-96, miR-888, miR330-3p, let-7a and miR-146a with all declining in adult blood. Among them, miR96 inhibits the expression of *γ globin* gene most potently. The amount of this miR expression has been more in adult blood reticulocytes that prevent γ globin mRNA by connect with the AGO2 in miRISC complex and plays an important role in post-transcriptional regulation of the expression of HbF during adult erythropoiesis (50). Several miR are able to increase *γ globin* gene expression such as Lin28B with let-7 family participating in the regulation of fetal to adult erythroid development process by increasing *γ globin* gene expression through inhibitory effects on BCL11A (53). BCL11A is one of the most significant regulators in switching between γ and β through γ gene silencing. MiR-486-3p binds to the 3'UTR of *BCL11A* and directly inhibits this factor which prevents it from γ gene silencing. MiR-486-3p thus plays a role in regulating the synthesis of HbF in adult erythropoiesis by inhibiting post-transcriptional regulation of *BCL11A* expression (14). 

In patients with trisomy 13, elevated levels of miR15a/16-1 results in additional down-regulation of *MYB* expression, a potent negative regulator of HbF expression, which in turn results in a delayed switch from fetal to adult hemoglobin and persistent expression of HbF. In other words, miR-15a/16-1 restrain the MYB factor which then cause loss of the inhibitory effect on γ gene and induce HbF in early erythroid progenitors (11). *MYB* may thus be seen as an important therapeutic target to increase HbF in patients with SCD and β-thalassemia. Consequently, these miRs may be used for the therapy of patients with hemoglobinopathies (4). 

Specificity protein (Sp)/KLF family of proteins contains three conserved zinc finger domains and has a regulatory role in erythroid differentiation and *globin* gene expression by binding to the CACCC/GC/GT boxes in the DNA (55). Two transcription factors, KLF3 and SP1 belong to the family of *β-like globin* gene transcription regulation that act by binding to the LCR regions of the ε, γ, and *β globin* promoters. Over expression of SP1 has been shown to reduce expression levels of ε and γ globin. Accordingly, this factor has been the main target for miR-23a which increases γ and ε globin expression by SP1 inhibition and repression. KLF3 factor, a negative regulator of erythropoiesis process (56), inhibits expression of γ globin and also the miR-23a cluster. MiR-27a is used to target the KLF3 factor which renders its negative effect on γ globin expression. Therefore, both miR-23a/27a have a remarkable ability to inhibit two negative regulators of the *globin* genes cluster and in turn up-regulate *β-like globin* genes (57). 

The kit receptor ligand (KL), capable of reactivating HbF synthesis in normal erythropoiesis, SCD and β-thalassemia, is involved in Hb switching with a direct connection between KL concentration and HbF synthesis (58). The main miRs central to the suppression of KL are miR-221/-222 which attach to the 3'UTR of kit and then reduce proliferation and differentiation of erythropoietic cells (59). These miRs reduce HbF production in erythropoiesis by targeting KL (58,60). Using exogenous KL or antagomir-221/-222 therapy could raise γ chain synthesis and therefore induce HbF production in patients dependent on HbF reactivation treatment (58). 

During the differentiation of hematopoietic progenitor cells to erythroid cluster and induction of hypoxia-inducible factor 1 (HIF1) in hypoxic conditions, miR-210 was shown to increase, expediting switching to *γ globin* gene by activating maturing erythroid progenitor cell transcription factors and instituting a link between hypoxia and erythropoiesis (28,61). The hypoxia-associated miR210 may therefore be a suitable target for HbF production and thus improving sickle cell disease and β-thalassemia (26,62). 

GATA1 is one of the important hematopoietic transcription factors in the production of blood cells, including platelets, eosinophils, mast cells and erythrocytes. During the embryonic period, it has a prominent role in the last stages of erythropoiesis by regulating genes involved in cell division, apoptosis leading to terminal maturation. Several miRs have been reported to regulate this factor during erythropoiesis (63). During erythroid maturation, miR-26b/-144/-451 have elevated expression and for the period of erythropoiesis can influence the expression of many genes of which one is *GATA1*. MiR-451 is more effective on α and *β globin* levels than γ while miR-26b increases expression of β and γ (64,65). Hydroxyurea, causing HbF augmentation in patients with hemoglobinopathies, is also effective on expression of miR-26b and miR-151 (54). Through these miRs, in the late stages of erythropoiesis, HbF synthesis may be enhanced. 

Hence, according to this survey, miRs contribute to the developmental progression of *globin* gene expression with some exclusively reactivating *γ globin* gene expression and subsequently HbF production. It is hoped that with elevated HbF levels, treatment of patients with β-hemoglobin disorders becomes more effective and better recovery is obtained. 

**Table 1:** Different roles of microRNA in γ globin expression


microRNA	Target(mRNAorprotein)	Biologicaleffect	Reference

Lin28B	BCL11A	Increasing *γ globin* gene expression	(66)
Let-7	γ globin mRNA	Suppressing γ globin expression	(50,67)
miR-15a/-16-1	*MYB* mRNA	Increasing *γ globin* gene expression	(11,33)
miR-23a/27a	*KLF3* and *SP1* mRNAs	Regulating *β-like globin* gene expression	(57)
miR-26b	*GATA1* RNA	Increasing *γ globin* gene expression	(64)
miR-96	CDS region of γ globin mRNA	Suppressing *γ globin* gene expression	(50)
miR-126	*MYB* mRNA	Decreasing *MYB* levels and suppressing erythropoiesis	(68)
miR-144	1. *GATA1* mRNA	1. Negatively regulating the α globin in embryonic erythropoiesis	(21,44,65,69)
	2. *KLFD* mRNA	2. Inducing of *γ globin* gene transcription	
	3. *NRF2* mRNA	3. Interference with antioxidant capacity; susceptibility to oxidative stress, hemolysis and severe anemia	
miR-146a	γ globin	Suppressing *γ globin* gene expression	(50)
miR-150	*MYB* mRNA	Suppressing α globin synthesis and erythropoiesis	(39,70)
miR-199-5p	c-Kit	Regulating of human erythropoiesis and decrease HbF levels	(71)
miR-210	To be identified	Increasing following erythroid differentiation and indirectly increase HbF	(28,61)
miR-221/-222	c-Kit	Decreasing of erythroblast proliferation and HbF levels	(58)
miR-451	1. GATA-1 and GATA-2 mRNAs	1. Inducing *γ globin* gene transcription and suppress α globin, Glycophorin-A	(35,65,72,73)
	2. 14–3-3ζ mRNA	2. inhibiting nuclear accumulation of FoxO3 transcription factors, a positive regulator of erythroid antioxidant genes	
miR-486-3p	BCL11A	Increasing expression of *γ globin* gene	(14)


miR; MicroRNAs, BCL11A; B-cell lymphoma/leukemia 11A protein, *MYB*; Proto-oncogene mRNA, *KLF3*; Krüppel-like factor 3 mRNA, *SP1*; Specificity protein 1 mRNA, *GATA1/2*; GATA-binding factor 1/2 mRNA, CDS region; Coding DNA Sequence region mRNA, *KLFD*; Krüppel-like factor d mRNA, *NRF2*; Nuclear factor (erythroid-derived 2)-like 2 mRNA, c-Kit; Tyrosine-protein kinase Kit or CD117 tyrosine-protein kinase Kit or CD117 protein and HbF; Fetal hemoglobin.

### Discussion

Among inherited blood disorders with defective hemoglobin production, mutations in human *HBB* gene is the most prevalent cause for SCD and β-thalassemia traits (74). In these disorders, enhancing the HbF level is a recompense mechanism to diminishing of clinical complications. Studies have revealed that HbF expression could be regulated post-transcriptionally. 

MiRs also have epigenetic actions by which they regulate post-transcriptional developmental processes including proliferation, differentiation, metabolism and apoptosis in different cells (4). Erythrocytes are not expected from this regulatory function as miRs of this cell type coordinate maturation and proliferation of early erythroid cells, expression of fetal *γ globin* genes and enucleation. In specific, miR-15a, miR-16-1, miR-126, miR-144, miR-451 and miR-210 affect erythroid cluster commitment with some of them increasing (miR-126/-188/210/-362/-451) and others decreasing (miR103/-150/-223/-376) during erythroid maturation (75). Moreover, miRs found to up regulate *γ globin* gene expression with their overexpression were miR-26b/-210/-451. In contrast, an appealing outcome on *γ globin* gene expression was found for miR-96 where its direct binding to γ globin transcript inhibited γ globin expression (76). Therefore, inhibition of this miR that has a suppressive effect may enhance HbF production. Consequently, miRs have the potential to enhance hematopoiesis in anemia and blood diseases. 

On the other hand, a lot of miRs are efficient in improving the situation of many cellular mechanisms. Sangokoya et al. (44), identified that high expression of miR-144 is associated with severity of anemia in SCD and may play a role in increasing oxidative damage and hemolysis in mature HbSS erythrocytes by affecting *NRF2* and oxidative stress tolerance. Transfecting anti-miR-144 into sickle cells may therefore reduce the levels of oxidative stress, relieving a major problem in these patients. Azzouzi et al. (50) not only assessed the direct effect of miR96 on the inhibition of *γ globin* gene expression, they also showed that inhibiting this miR can be used to induce HbF. 

## Conclusion

Acording to studies on cell lines and identification of the roles played by miRs in erythroid differentiation and HbF production, it is recommended to target pharmacological miR inhibitors of HbF through the use of antisense molecules or use exogenous miRs that stimulate HbF production as a novel diagnostic/prognostic strategy. Although further studies may be required to achieve this goal, this approach has the potential to become more successful in treating patients with β globin disorders. 
